# Supplementary material for: Transcriptional Regulation of Structural and Functional Adaptations in a Developing Adulthood Myocardium
Source: Cardiol Cardiovasc Med. Author manuscript; Available in PMC 2021 Sep 7. (PMC8423094; doi:10.26502/fccm.92920215)
Supplement: 1 [file NIHMS1719618-supplement-1.pdf]

## Supplementary Table

| Gene Symbol | Forward | Reverse |
|---|---|---|
| Gsta1 | AGCCCGTGCTTCACTACTTC | TCTTCAAACTCCACCCCTGC |
| Acta2 | CCCTGGAGAAGAGCTACGAAC | TTTCGTGGATGCCCGCTG |
| Timp3 | GAAGCCTCTGAAAGTCTTTGTGG | ACATCTTGCCTTCATACACGC |
| Timp4 | TGTGGCTGCCAAATCACCA | TCATGCAGACATAGTGCTGGG |
| c1s1 | GGCCCACTTGTTCCCATCAG | TTGGTAGTGAGGGACCACCC |
| Myh6 | GAGTGGGAGTTTATCGACTTCG | CCTTGACATTGCGAGGCTTC |
| Arbp1 | GAGATTCGGGATATGCTGTTGG | CGGGTCCTAGACCAGTGTTCT |
| nppb | GAGGTCACTCCTATCCTCTGG | GCCATTTCCTCCGACTTTTCTC |
| aldob1 | GAAACCGCCTGCAAAGGATAA | GAGGGTCTCGTGGAAAAGGAT |
| mmp9 | GCTGACTACGATAAGGACGGCA | TAGTGGTGCAGGCAGAGTAGGA |
| mmp2 | AGCGAGTGGATGCCGCCTTTAA | CATTCCAGGCATCTGCGATGAG |
| Gapdh | TGACCTCAACTACATGGTCTACA | CTTCCCATTCTCGGCCTTG |

**Table S1:** Complete list of primer sequences used for qPCR analysis.
